# Antidepressant Use During Breastfeeding

**DOI:** 10.2174/157340411794474784

**Published:** 2011-02

**Authors:** Jan Øystein Berle, Olav Spigset

**Affiliations:** 1 Department of Psychiatry, Haukeland University Hospital, P.O. Box 23 Sandviken, N-5812 Bergen, Norway; 2 Department of Clinical Pharmacology, St Olav University Hospital, Trondheim, Norway; 3 Department of Laboratory Medicine, Children’s and Women’s Health, Norwegian University of Science and Technology, Trondheim, Norway

**Keywords:** Antidepressant, breastfeeding, postpartum, nursing infant, SSRI, depression.

## Abstract

**Background:**

The treatment of breastfeeding mothers with depression raises several dilemmas, including the possible risk of drug exposure through breast milk for the infant. This article provides background information and presents practical advice and recommendations for the clinician dealing with the treatment of depression and related disorders in the postpartum period.

**Methods:**

An electronic search for relevant articles was performed. As the use of tricyclic antidepressants has considerably decreased during the last decade and no new information on breastfeeding has emerged for the tricyclics in this period, this review exclusively focuses on the newer, non-tricyclic compounds.

**Results:**

Most newer antidepressants produce very low or undetectable plasma concentrations in nursing infants. The highest infant plasma levels have been reported for fluoxetine, citalopram and venlafaxine. Suspected adverse effects have been reported in a few infants, particularly for fluoxetine and citalopram.

**Conclusions:**

Infant exposure of antidepressants through breast milk is generally low to very low. We consider that when antidepressant treatment is indicated in women with postpartum depression, they should not be advised to discontinue breastfeeding. Paroxetine and sertraline are most likely suitable first-line agents. Although some concern has been expressed for fluoxetine, citalopram and venlafaxine, we nevertheless consider that if the mother has been treated with one of these drugs during pregnancy, breast-feeding could also be allowed during continued treatment with these drugs in the postpartum period. However, an individual risk-benefit assessment should always be performed.

## INTRODUCTION

Depression is a common illness among women in the postpartum period; a prevalence of 10 to 15 % is often reported [1-3]. During this period, challenges are related to caretaking of the newborn infant in addition to the potentially harmful effects of the depression on the mother. Depressed mothers may be intrusive or withdrawn and disengaged, and are less sensitively attuned to their infants than healthy women [4]. In a study of 112 mother-infant pairs, chronic maternal depression in the first year postpartum was related to delayed psychomotor development in the infant at 15 months [5]. Moreover, untreated maternal depression may also affect the cognitive and emotional development of the infant. Thus, the high prevalence of postpartum depression, causing functional impairment in the mother and potential disturbances of the mother-infant relationship, makes it important to initiate effective, rapid-onset therapies in women suffering from this disorder [4, 6-8].

Postpartum anxiety disorders are underemphasized and may be even more common than postpartum depression [6]. The peak age of onset for anxiety disorders in women corresponds with their childbearing years, and particularly the rates of obsessive-convulsive disorder and generalized anxiety disorder are increased in postpartum women. Due to few studies only very limited data are available to guide clinical interventions for women with or at risk for having perinatal anxiety disorders [9]. Medication with antidepressants, in particular selective serotonin reuptake inhibitors, may be indicated also in some of these women. 

The benefits of breastfeeding are well documented, both for the infant and the mother [7]. Human milk represents the ideal primary source of nutrients and provides better immunological and antioxidant protection than do milk substitutes [7, 10, 11]. Therefore, women are strongly encouraged to breastfeed when possible [12]. Both the American Academy of Pediatrics and the World Health Organisation recommend the exclusive use of breast milk for 6 months, with use of milk substitutes only for infants who cannot be breastfed [12, 13].

The dilemma in the treatment of breastfeeding mothers is weighing the potential risk to the infant of antidepressant exposure through breast milk against the disadvantage of not receiving mother’s milk. A third alternative, to discontinue or not commence drug treatment, might be even more harmful, taking into account the risk of not receiving adequate treatment for the mother and thereby indirectly also for the infant [5, 6]. Specific questions to be answered when deciding how to handle a woman with postpartum depression include: What are the risks for the mother and the infant if the maternal depression is not adequately treated? How strong is the mother’s desire to breastfeed her infant? What are the disadvantages of not receiving mother’s milk for the infant? What are the risks for the infant of being exposed to an antidepressant through breast milk? Is there any evidence to suggest that some antidepressants are more favorable than others to use during breastfeeding, and are there sufficient data to give conclusive advice for the most recently marketed antidepressants? Could any practical strategies be used to reduce the drug exposure to the infant? And finally, given that there is a (small) risk of adverse effects in the infant due to drug exposure and breastfeeding nevertheless is allowed, should the infant be monitored in any way?

In some cases, non-pharmacological treatment may be an option, and women with postpartum depression tend to prefer non-pharmacological treatment instead of using medicines [14]. It has also been shown that women in the postpartum period receive fewer prescriptions of psychotropic drugs than do non-breastfeeding women, but although psychotherapy is effective in the treatment of postpartum depression [15], it is not widely available. Thus, there is a risk that women not receiving antidepressant treatment would be inadequately treated for their illness. One major study showed that psychological intervention for post-partum depression improved maternal mood in the short term, but that this benefit was not superior to spontaneous remission in the long run [16]. 

A few studies have specifically addressed the effect of antidepressants in the postpartum period. Recent case reports, case series and open trials suggest efficacy in women suffering from postpartum depression, although many of these trials excluded women who were breastfeeding [14]. Several studies have shown improvements in postpartum depressive symptoms resulting from treatment with selective serotonin reuptake inhibitors (SSRIs), such as sertraline [17], fluvoxamine [18] and fluoxetine [19], and the SSRIs are thus considered first-line therapy in postpartum depression [20]. The SSRI group is also recommended in the treatment of postpartum dysthymia, panic disorder, and obsessive-compulsive disorder [21]. In addition, the serotonin-noradrenaline reuptake inhibitor (SNRI) venlafaxine has been found to reduce the symptoms of postpartum depression [22]. 

Women with a previous episode of postpartum depression comprise a high-risk group for subsequent episodes; a recurrence risk of about 25% has been reported [23]. The findings from a small randomised study comparing sertraline and placebo in asymptomatic women with at least one prior episode of postpartum depression suggest that postpartum depression can be prevented [24], although the result needs to be replicated in a larger scale. Psychosocial or psychological interventions have not been shown to significantly prevent the risk of developing postpartum depression [25]. 

A Cochrane review of antidepressant prevention of postpartum depression from 2005 concluded that it is not possible to draw any clear conclusions about the effectiveness of antidepressants in preventing postpartum depression [26]. The reason was the lack of conclusive evidence, and the authors stated that larger trials were needed. 

In some cases, there is a question of whether an effective antidepressant treatment given during pregnancy could be continued or not when the mother wants to breastfeed. Discontinuing essential antidepressant treatment in the postpartum period should be avoided, and switching to another antidepressant might also be problematic in this vulnerable period. Thus, the issue of infant safety in the postpartum period should preferably be taken into consideration already when drug treatment is started in a woman, irrespective of whether it is before or during pregnancy. 

Knowledge about infant effects of antidepressants transferred *via* breast milk is mostly based upon case studies and small case series. A comprehensive review and pooled analysis of antidepressant levels in breast milk and nursing infants, including possible adverse effects in the infants, was published in 2004 [27]. An update of this review, adding new information from the period 2004 - 2008, was published in 2009 [7]. The aim of the present article is to take even newer data into account, providing aggregated background information and presenting practical advice and recommendations for the clinician dealing with the treatment of depression in the postpartum period. During the last decade, the use of tricyclic antidepressants has considerably decreased, mostly because they are no longer considered first-line therapy due to their adverse effect profile and toxicity. Therefore, this review focuses on the newer, non-tricyclic antidepressants, i.e. the SSRIs citalopram, escitalopram, fluoxetine, fluvoxamine, paroxetine and sertraline, the SNRIs venlafaxine and duloxetine, and reboxetine, bupropion and mirtazapine. First, we present data on the levels of these antidepressants in breast milk and in infant plasma; thereafter we discuss the reported short-term and long-term adverse effects in infants. Finally, we suggest a number of practical recommendations.

## ANTIDEPRESSANT LEVELS IN BREAST MILK

Detectable levels have been found in breast milk for all antidepressants studied [7, 27-29]. In general, drug concentrations in milk parallel those in maternal plasma, but with a slight delay. Due to the lipophilicity of the drugs, milk levels are typically somewhat higher than the levels in maternal plasma. For example for citalopram, the milk/plasma concentration ratio, i.e. the ratio between the concentration in milk and in maternal plasma, is in the interval 1.2–3.0 [10, 30]. Notably, the triglyceride levels in milk have been shown to influence the drug concentrations. In a study from our group, higher triglyceride levels were, as expected, found in post-feed milk than in pre-feed milk, with accordingly higher drug concentrations in post-feed milk, as shown e.g. for citalopram, sertraline and fluoxetine [10]. Consequently, for research purposes and when comparing results from different studies, it is important to provide information about in which phase the milk sample has been obtained. 

The milk drug concentration can be used to estimate the daily drug dose ingested by the infant, assuming an average milk intake of 150 ml per kg body weight per day. The infant dose per kg body weight can then be expressed as a percentage of the maternal dose per kg body weight. When the relative dose is below 10%, the exposure could generally be considered negligible, and a notational level of concern of 10% has therefore been suggested [31]. The 10% limit has subsequently also been accepted by organisations such as the American Academy of Pediatrics. The relative infant doses are close to 10% and in some cases even above 10% for citalopram, fluoxetine and venlafaxine, and somewhat lower for escitalopram, whereas the relative infant doses are low for fluvoxamine, paroxetine, sertraline, duloxetine, reboxetine, bupropion and mirtazapine (Table **1**).

## ANTIDEPRESSANT LEVELS IN THE INFANT

Obviously, the drug concentration in infant plasma is a more direct measure of infant exposure than is the milk concentration. Among the SSRIs, paroxetine, fluvoxamine and sertraline basically produce undetectable plasma levels. The levels of citalopram have been measurable in some infants, albeit mostly relatively low. Fluoxetine and venlafaxine produce the highest infant plasma concentrations [10, 27], in some infants up to more than 80% and 30% of the assumed therapeutic concentrations in adults, respectively. However, such high concentrations are uncommon; e.g. for fluoxetine, infant plasma concentrations higher than 10 % of the maternal plasma levels were found in 8 of 36 cases (22 %), only, in a review [27]. The other antidepressants have either been undetectable (duloxetine, bupropion) or been detected at extremely low concentrations (escitalopram, reboxetine, mirtazapine) in infant plasma (Table **1**).

It should be noted that in the cases where high concentrations have been found, the infants have generally been below 3-4 months of age [32]. The capacity to metabolize drugs is generally not fully developed in neonates, but increases gradually as the hepatic function matures during the first 3-6 months postpartum. After this period, measurable infant plasma levels would not be expected to occur for any antidepressant. On the other hand, in preterm newborns, the metabolic capacity will be even more impaired than in full-term neonates. As a consequence of the immature hepatic function in neonates and the gradual development of the metabolic capacity of drugs, infant age is a central factor to take into account when performing an individual risk analysis.

Genetically determined differences in the metabolic capacity of hepatic enzymes involved in the metabolism of antidepressants may also affect the infant plasma levels. The most relevant enzymes with regard to antidepressant metabolism are the polymorphic cytochrome P-450 enzymes CYP2D6 and CYP2C19. In a study of 24 lactating mothers treated with SSRIs or venlafaxine and their 25 infants, a genetically determined impaired capacity to metabolise drugs *via* these enzymes (in the mother and/or the infant) did not significantly increase infant exposure [10]. Notably, a mother who was a CYP2C19 poor metaboliser and was treated with the CYP2C19 substrate citalopram had the highest plasma concentration of citalopram among all subjects studied. Nevertheless, her twin infants (who both were heterozygous extensive CYP2C19 metabolisers) still had extremely low plasma concentrations of citalopram [10].

## POSSIBLE ADVERSE EFFECTS IN THE INFANT

Whether the low antidepressant concentrations found in most infants are able to exert pharmacological effects remains an open question. For the drugs causing the lowest – and in many cases even undetectable – levels, such an influence is unlikely. In two studies attempting to elucidate the pharmacodynamic potential of antidepressants in breastfed infants, platelet serotonin levels were measured in the infants before and during maternal treatment with SSRIs [33, 34]. In the first study, no differences were observed when the mothers were treated with sertraline [33]. In the second study, no effects were seen in 10 of 11 infants whose mothers were treated with fluoxetine [34].

Adverse events in breastfed infants exposed to newer antidepressants through breast milk have been suspected in a few cases, and more often after exposure to fluoxetine and citalopram than after exposure to other drugs [7, 27, 35-40]. The effects observed have mostly been subtle and unspecific, and may even have arisen by coincidence. For example, crying, irritability, decreased feeding and watery stools have been described in a few cases for fluoxetine [35-37]. For citalopram, hypotonia, colic, decreased feeding and sleep difficulties have been reported in single cases [38, 39]. For other SSRIs the observations are even scantier [40]. Although single case reports are difficult to interpret with regard to causality it is interesting to note that the number of reports is higher for the drugs showing the highest infant plasma levels, i.e. citalopram and fluoxetine. For venlafaxine, which also has a relatively high infant exposure, no adverse events have been reported [10, 41-43], but the total number of exposed infants is considerably lower for venlafaxine than for e.g. citalopram and fluoxetine (Table **1**).

A single case of seizures has been reported in a 6-month-old breastfed infant after 4 days of maternal bupropion treatment [44]. Although seizures are a well-known adverse effect of bupropion, the relationship remains obscure, particularly as the infant was relatively old and had a respiratory tract infection, and as the levels of bupropion and/or active metabolites were not documented, neither in milk nor in the infant. 

Based on known adverse reactions after therapeutic use of SSRIs and previously suspected adverse events in breastfed infants we developed a questionnaire of infant symptoms to be filled in by the mothers [10]. The questionnaire was completed by 20 mothers treated with SSRIs or venlafaxine and well as by a control group of 68 medication-free breastfeeding mothers. The questionnaire included signs such as sneezing, regurgitation or vomiting, loud crying, decreased sleep, increased sleep, irritability, tremor, decreased muscle tone, and suckling or feeding problems. There were no significant differences in any symptoms between the two groups [10]. 

Another study has compared the frequency of possible adverse events in 31 infants whose mothers were treated with citalopram, 7 infants whose mothers were treated with other SSRIs, 5 infants whose mothers were treated with non-SSRIs and 31 infants of healthy mothers not taking any medication [39]. There were no significant differences in the frequency of signs reported in the infants between the groups (3/31, 0/12 and 1/31, respectively). Nevertheless, the three infants in the citalopram group presented with decreased feeding, colic and irritability, respectively, which have been suspected as adverse effects also in other reports [38, 39].

A specific safety index for antidepressant use in breastfeeding mothers has been proposed [40]. The index is expressed as the ratio between the reported number of infants with adverse events after exposure to an antidepressant through milk and the reported total number of exposed infants for the same antidepressant, multiplied by 100. It is suggested that a value ≤2 indicates that the drug is relatively safe; a value of 2.1-10 indicates that the drug should be used with great caution, and a value above 10 indicates that the drug should be contraindicated in breastfeeding mothers. The index has some limitations, such as that it is less reliable when the number of cases in the literature is low, and that it does not take into consideration the quality of raw data, e.g. whether the suspected adverse events most likely have arisen by coincidence or whether the relationship to drug exposure more likely is causal. Finally, there is a risk that the numbers found are abnormally high, as one could expect that there are numerous exposures without any adverse effects that have not been reported in the literature, whereas the reporting rate is considerably higher for infants where possible adverse effects were observed. By applying the safety index on the cases reported before 2007, the calculated values were 0.68 and 0.95 for sertraline and paroxetine, and 3.5 and 5.3 for fluoxetine and citalopram, respectively [40]. These differences in safety index values between the various drugs are consistent with what would be expected from literature reviews applying more qualitative investigative approaches [7, 27]. 

In conclusion, even though antidepressants probably have been subject to more published data then any other drug class (cf. Table **1**) there is, with a few exceptions, little evidence for causality between exposure through breast milk and adverse events in the infants [45].

## THE POTENTIAL RISK OF LONG-TERM EFFECTS

Several studies have investigated the possible effect of SSRI exposure *via* breast milk and body weight increase during the first year of life. In a study of 78 nursing infants whose mothers were treated with SSRIs (n=75) or venlafaxine (n=3), there were no differences in body weight compared to values from normative populations [46]. In a study on 11 infants exposed to citalopram, there were no differences in body weight after 12 months compared to 10 infants of medication-free mothers [47]. Finally, in a study on 27 infants exposed to paroxetine, there were no differences in body weight at 6 and 12 months compared to 27 breastfeed and 19 bottle-feed infants of medication-free mothers [48].

Long-term nevrobehavioural data on infant antidepressant exposure through breast milk is generally lacking. Such studies are also methodologically challenging, as the longer follow-up time, the greater influence would other factors, such as maternal mental health, be expected to have on the infant, relative to the role of drug exposure through breast milk. Although there is a lack of data concerning exposure through breast milk, some long-term information is available after antidepressant exposure in utero [47-52]. In these studies, no detrimental long-term effects were revealed for factors such as global intelligence quotient, language, behavioural development and neurological development. In a recent study in four-year-old children investigated by the Child Behaviour Check List [53], maternal mood was found to be more predictive of internalising behaviours than prenatal exposure to psychotropic drugs. Moreover, in another study no differences in externalising behaviours were detected between four-year-old children exposed to SSRIs during pregnancy and a non-exposed group [54]. These results are reassuring, as antidepressant exposure in utero causes serum concentrations that are at least 5- to 10-fold higher than exposure through breast milk [47, 52, 55]. 

## CLINICAL RECOMMENDATIONS

Several attempts to guide clinical decisions regarding breast-feeding during treatment with antidepressants have been made, including some more comprehensive reviews and overviews [7, 27, 29, 40, 45]. Also scientific organisations have attempted to develop practical guidelines [56-58], but two of these guidelines [56, 58] put more focus on drug treatment during pregnancy than in the postpartum period and are relatively vague with regard to the choice of specific drugs in lactating women. 

Most of the reviews and guidelines recommend that the choice of specific treatment should be based upon an individual risk-benefit analysis. In such an analysis both the risk of untreated maternal illness for the mother and the infant, the risk/benefit of the specific treatment for the mother and the infant, the risk/benefit of being breastfed or not for the infant, the possible maternal risks of renouncing breastfeeding, and the mother’s desire to breastfeed should be taken into consideration. Medication exposure may involve a risk for the infant, but there are also risks both with an untreated depression and of not receiving mother’s milk for the infant. Therefore, no clinical decision in the context of postpartum depression is completely risk-free [59].

Non-pharmacological interventions such as psychotherapy should be considered, particularly for mild to moderate depression. For women with moderate to severe depression and in some cases also with anxiety disorder, medications are generally the most appropriate choice of treatment. Moreover, for women with previous postpartum depression or women who have been treated with antidepressants during pregnancy, antidepressants are the preferred mode of treatment in the prophylaxis or new episodes/relapses. 

Regarding the choice of specific antidepressant, it is usually recommended that paroxetine and sertraline should be preferred over other SSRIs due to the low infant exposure for these drugs (Table **1**) [7, 29, 40, 57]. However, paroxetine might have some disadvantages related to the treatment of women in fertile age in general. First, if the mother needs long-term treatment and subsequently becomes pregnant once again, paroxetine is probably not the first choice due to the risk of cardiac defects. Second, the risk of withdrawal symptoms might be higher than for other SSRIs if one or a few doses are missed (which possibly might occur more often among busy, and perhaps even sleep-deprived, post-partum mothers than among other women).

It is often recommended that when possible, fluoxetine and citalopram should be avoided or used with caution due to the higher infant plasma levels than for other drugs and the possible risk of adverse effects in the infant [7, 29, 40]. However, if the mother has been treated with fluoxetine or citalopram previously and the treatment was effective, or if the mother has used one of these drugs during pregnancy, it could also be used in the postpartum period [7, 29]. In a study from our group including various SSRIs and venlafaxine [10], we concluded that when antidepressant treatment is indicated in the postpartum period, the women should generally not be advised to discontinue breastfeeding.

The numbers of exposed cases vary significantly between drugs, with about 100 cases for fluoxetine, paroxetine, sertraline and citalopram, but less than 25 for the other newer antidepressants (Table **1**). Some degree of uncertainty inevitably exists for the drugs with the lowest numbers of exposed infants, also when no adverse effects have been reported. On this basis, drugs for which little data exist, such as fluvoxamine, venlafaxine, duloxetine, reboxetine, bupropion and mirtazapine, should not be considered as first-line therapies, but they can be used in special cases [7, 29, 57]. 

Some of the reviews and guidelines recommend infant monitoring, particularly if the infant is sick, premature or has a low body weight [7, 27, 57]. However, given the very low risk of infant effects and the unspecific nature of possible infant symptoms, we consider there is no general need for regular and specific follow-up examinations [29]. 

Routine breast milk and/or infant serum sampling for drug concentration analysis is generally not recommended [7, 27, 45]. It can, however, be helpful if the infant has signs that may be indicative of drug exposure [57]. In addition, it is our experience that it is often reassuring for a mother who has a strong desire to continue breastfeeding to know that the drug is found in negligible concentrations in milk and/or infant plasma. Thus, arguments exist to perform milk or infant plasma drug analyses more liberally than often recommended, at least when such analyses are easily available.

As antidepressants are lipophilic drugs, their excretion in breast milk is expected to vary with milk triglyceride content [10, 60]. Yet, the nutritional value of human milk is also linked to its triglyceride levels. Therefore, any effort to avoid the rather minimal additional drug exposure imposed by breast milk containing high *vs*. low triglyceride levels cannot be recommended [10].

Most antidepressants have long elimination half-lives and their levels in breast milk generally vary relatively little during a dose interval. Consequently, avoiding breastfeeding during the peak concentration phase, e.g. by taking the daily drug dose in the evening and avoiding breastfeeding during the night, will only reduce the infant drug intake to a small extent. 

Pumping and discarding breast milk to reduce the exposure is of little value. First, there is no indication that there is a risk threshold that is crossed if the infant exposure is reduced to some extent, e.g. by 30-50%. Second, discarding breast milk implicitly implies that drug exposure is detrimental if the infant is feeded by breast milk, only. Third, in our experience, a procedure of e.g. 50% breast-feeding and 50% bottle-feeding most often ends with bottle-feeding, only, after a relatively short period of time.

## CONCLUSION

Postpartum depression is a potentially serious condition in need of effective treatment. Several modalities of intervention may be helpful, and both psychotherapy and treatment with antidepressants are recommended. 

Data from a large number of studies show that antidepressants differ with respect to infant exposure. Available data are clearly homogenous with this respect, showing that in the SSRI group, paroxetine and sertraline are excreted in milk in low amounts that do not produce measurable concentrations in infant plasma, and these drugs have neither been associated with clear-cut adverse effects in the infant. On the other hand, fluoxetine and citalopram are excreted in milk in higher amounts, which in a few cases have caused significant plasma levels in the infant and suspected adverse effects. For other newer antidepressants, data are scarce, with relatively few exposed infants reported. Of these drugs, preliminary data indicate that venlafaxine causes somewhat higher infant exposure than the other drugs.

Taking all current knowledge into consideration, we suggest that when antidepressant treatment is indicated in women with postpartum depression, they should generally not be advised to discontinue breastfeeding. With regard to choice of specific agent, paroxetine and sertraline should be considered first. Nevertheless, although some concern has been expressed for fluoxetine, citalopram and venlafaxine, we consider that if the mother has been treated with one of these drugs during pregnancy, breast-feeding could generally also be allowed during continued treatment with these drugs instead of switching to a “safer” drug. However, these drugs, in addition to drugs for which very little information exist, should not be considered first-line agents if there are not specific reasons for preferring them.

## Figures and Tables

**Table 1:** Infant Doses and Plasma Concentrations of Newer Antidepressants after Excretion in Breast Milk

Drug	Approximate Number of Mother/Infant Pairs Studied^1^	Absolute Infant Dose (mg/d)^2^	Relative Infant Dose (%)^3^	Absolute Infant Plasma Concentrations (ng/ml)	Relative Infant Plasma Concentrations (%)^4^
**Selective serotonin reuptake inhibitors**					
Citalopram	80^12^	0.14	3-10	Negligible^5^	Up to 10^6^
Escitalopram	12	0.04	3-6	<5	<4
Fluoxetine^7^	149	0.14	<12	Up to 100^8^	Up to 80^8^
Fluvoxamine	12	0.12	<2	Not detected^9^	-
Paroxetine	119	0.03	0.5-3	Not detected^9^	-
Sertraline	145	0.04	0.5-3	Not detected^9^	-
**Other antidepressants**					
Venlafaxine^10^	23^13^	0.50	6-9	Up to 40	Up to 30
Duloxetine	6^14^	<0.03	<1	Not detected 9	-
Reboxetine	4	0.03	1-3	<5	<2
Bupropion^11^	20^15^	0.20	2	Not detected^9^	-
Mirtazapine	11^16^	0.04	0.5-3	0.2 16	<1 16

1 The numbers given here are the sum of the number of cases included in the review by Weissmann *et al.* [27], the number of cases included in the review by Lanza di Scalea *et al.* [7], and the number of cases obtained from literature published after the Lanza di Scalea review was completed. The new references are shown in footnotes 12-16.

2 Calculated for an infant with a body weight of 5 kg, and assuming a daily milk intake of 150 ml/kg body weight.

3 Infant daily dose per kg body weight expressed as a percentage of maternal daily dose per kg body weight. A value below 10 % is generally considered negligible.

4 Infant plasma concentration expressed either as a percentage of the measured maternal plasma concentration or as a percentage of what could be considered a low therapeutic concentration in adults [61].

5 In most cases below the lower limits of detection for the analytical methods employed, which were mostly in the range of 2–5 ng/ml. However, in a few cases, which also have been associated with suspected adverse effects, concentrations up to 15 ng/ml have been found.

6 In a few cases, which also have been found associated with suspected adverse effects, concentrations up to about 50 % of the therapeutic concentration range have been found.

7 The values represent the sum of fluoxetine and the active metabolite norfluoxetine.

8 In some cases, which also have been associated with suspected adverse effects, as high as about 500 ng/ml, i.e., clearly within the therapeutic concentration range.

9 Below the lower limits of detection for the analytical methods employed, which were mostly in the range of 1–5 ng/ml.

10 The values represent the sum of venlafaxine and the active metabolite O-desmethylvenlafaxine.

11 Including one or several of the active metabolites of bupropion.

12 One new case (cf. footnote 1) is obtained from Werremeyer, 2009 [62].

13 Thirteen new cases (cf. footnote 1) are obtained from Newport *et al.*, 2009 [43].

14 One new case (cf. footnote 1) is obtained from Briggs *et al.*, 2009 [63].

15 Four new cases (cf. footnote 1) are obtained from Davis *et al.*, 2009 [64].

16 One new case (cf. footnote 1) is obtained from Tonn *et al.*, 2009 [65]. In this case, the infant plasma concentration was 10 ng/ml, corresponding to about 30 % of the maternal plasma concentration.
